# Perceptions of health stakeholders on task shifting and motivation of community health workers in different socio demographic contexts in Kenya (nomadic, peri-urban and rural agrarian)

**DOI:** 10.1186/1472-6963-14-S1-S4

**Published:** 2014-05-12

**Authors:** Beverly Marion Ochieng, Edith Akunja, Nancy Edwards, Diana Mombo, Leah Marende, Dan CO Kaseje

**Affiliations:** 1 Great Lakes University of Kisumu P. O. Box 2224-40100 Kisumu, Kenya; 2 University of Ottawa, Canada

**Keywords:** Task shifting, motivation, peri-urban, nomadic, rural, Community Health, Community Health Workers, délégation de tâches, motivation, périurbain, nomade, rural, santé communautaire, travailleurs en santé communautaire

## Abstract

**Background:**

The shortage of health professionals in low income countries is recognized as a crisis. Community health workers are part of a “task-shift” strategy to address this crisis. Task shifting in this paper refers to the delegation of tasks from health professionals to lay, trained volunteers. In Kenya, there is a debate as to whether these volunteers should be compensated, and what motivation strategies would be effective in different socio-demographic contexts, based type of tasks shifted. The purpose of this study was to find out, from stakeholders’ perspectives, the type of tasks to be shifted to community health workers and the appropriate strategies to motivate and retain them.

**Methods:**

This was an analytical comparative study employing qualitative methods: key informant interviews with health policy makers, managers, and service providers, and focus group discussions with community health workers and service consumers, to explore their perspectives on tasks to be shifted and appropriate motivation strategies.

**Results:**

The study found that there were tasks to be shifted and motivation strategies that were common to all three contexts. Common tasks were promotive, preventive, and simple curative services. Common motivation strategies were supportive supervision, means of identification, equitable allocation of resources, training, compensation, recognition, and evidence based community dialogue.

Further, in the nomadic and peri-urban sites, community health workers had assumed curative services beyond the range provided for in the Kenyan task shifting policy. This was explained to be influenced by lack of access to care due to distance to health facilities, population movement, and scarcity of health providers in the nomadic setting and the harsh economic realities in peri-urban set up. Therefore, their motivation strategies included training on curative skills, technical support, and resources for curative care. Data collection was viewed as an important task in the rural site, but was not recognized as priority in nomadic and peri-urban sites, where they sought monetary compensation for data collection.

**Conclusions:**

The study concluded that inclusion of curative tasks for community health workers, particularly in nomadic contexts, is inevitable but raises the need for accreditation of their training and regulation of their tasks.

## Abstract (fr)

**Résumé:**

**Contexte**

La pénurie de professionnels dans les pays à faible revenu constitue une crise. Les travailleurs en santé communautaire font partie d’une stratégie de « délégation de tâches » visant à résoudre cette crise. La « délégation de tâches » consiste à confier à des bénévoles formés certaines tâches incombant habituellement aux professionnels de la santé. Au Kenya, on se demande si les bénévoles doivent recevoir une forme de compensation et quelles seraient les stratégies de motivation efficaces en fonction du contexte sociodémographique et du type de tâche déléguée. Cette étude visait à recueillir les points de vue de divers intervenants à l’égard des types de tâches à déléguer aux travailleurs en santé communautaire et des stratégies appropriées pour les motiver et les maintenir en poste.

**Méthodes**

L’étude a pris la forme d’une analyse comparative fondée sur des méthodes qualitatives, à savoir, des entrevues réalisées auprès d’intervenants clés (notamment des responsables des politiques de santé, des gestionnaires et des fournisseurs) et des groupes de discussion réunissant des travailleurs de la santé et des prestataires de services visant à connaître leur opinion sur les tâches déléguées et sur les stratégies de motivation appropriées.

**Résultats**

L’étude a révélé que certaines tâches à déléguer et stratégies de motivation étaient les mêmes dans les trois contextes démographiques. Les tâches communes aux trois contextes étaient la promotion, la prévention et les services curatifs simples. Les stratégies de motivation communes aux trois contextes étaient la supervision empreinte de soutien, les outils de dépistage, l’affectation équitable des ressources, la formation, la rémunération, la reconnaissance et un dialogue communautaire fondé sur des données probantes.

L’étude a en outre montré qu’au sein des populations nomades et périurbaines, les travailleurs en santé communautaire assument des tâches curatives qui dépassent la portée de la politique de délégation de tâches en vigueur au Kenya. Cette situation est attribuable au fait que l’accès aux soins est difficile compte tenu : de la distance que doivent parcourir certaines populations pour atteindre les établissements de santé; des mouvements des populations nomades et de la rareté des fournisseurs de soins de santé au sein de ces mêmes populations; de la réalité économique difficile dans les milieux périurbains. Les stratégies de motivation pour ces populations comprenaient : la formation aux tâches curatives; le soutien technique; l’affectation de ressources aux fins des traitements. L’étude a aussi révélé que la collecte de données, si elle est vue comme une tâche importante au sein des populations rurales, n’est pas jugée prioritaire par les populations nomades et périurbaines, qui réclament en échange une compensation financière.

L’étude a permis de conclure que l’ajout de tâches curatives aux fonctions des travailleurs en santé communautaire, particulièrement au sein des populations nomades, est inévitable, mais qu’il appelle la certification de leur formation et la réglementation de leurs tâches.

## Background

Task shifting refers to the rational redistribution of tasks from health professionals to community health workers in order to improve access to care and optimize the use of limited human resources. Through task shifting, the impact of health worker shortfalls may be mitigated [1,2]. Community Health Workers, that are trained lay volunteers, are an important part of a “task-shift” strategy and may help address the growing crisis of health worker shortages [3,4].

Community Health Workers (CHWs) have contributed to improved access to and coverage of health services in remote areas leading to improved health indicators [3]. According to World Health Organization (WHO) report “Working together for health,” there have been innumerable experiences with CHWs throughout the world with programmes ranging from large-scale, national programmes to small-scale, community-based initiatives [5]. In many countries, CHWs have acted as a bridge between formal health systems and the community [4], enabling health programmes to achieve three interconnected goals: building a relationship between the health care provider and the community; improving appropriate health care utilization; and educating people to reduce health risks in their lives [6].

In Kenya, CHWs have been trained by the Ministry of health and non-governmental organizations to carry out health tasks since the 1970s [7]. Since that time there have been a number of initiatives in many parts of the country, mainly by non-governmental organizations. In 2006, the Kenyan government launched a national program involving the training and deployment of CHWs as part of the community health strategy to reach every household with an essential care package for the community level, as provided for in the health sector strategic plan II [8], and the norms and standards [8].

In Kenya, CHWs are men and women who are literate and must come from the communities they serve. They are selected by their own communities. Keeping them as informal service providers, supporting home care givers, ensures that they remain embedded in their local context, sympathetic to local views and aspirations.

Although there is strong research evidence demonstrating the effectiveness of CHWs, retention challenges have been reported by many researchers [9,10]. The most common reasons cited include lack of long-term career opportunities, informal training with no formal certification and no transferable qualifications, professional protectionism and government interest in training and retention of CHWs that ebbs and wanes [9,10]. Task shifting has been given particular urgency in the face of demands placed on health systems, particularly in low income settings with both high burden of communicable and non-communicable diseases [1,9,11,12] Sustainable and effective motivational strategies are critical components of any package of interventions designed to retain health workers [13]. Franco et al [14] have argued that while workers often state that financial incentives are the key to motivation, there are a number of non-financial interventions that may be more effective means of improving performance.

There is evidence that CHWs play an important role in helping to achieve the Millennium Development Goals for health, particularly for child survival and treatment of tuberculosis (TB) and HIV/AIDS [1,11,15-19]

Shifting the information gathering tasks to CHWs could improve the timeliness of data, if analyzed and used close to collection sites, and thus increase local demand for information. Researchers have suggested that applying a population based information system that permits timely availability of information can improve the performance of the health system [20,21]. Health Information Systems offer opportunities to inform health decision making at all levels of the health system [22]. Communities require appropriate health information to choose healthy behaviours, to demand effective services, and to hold their governments accountable for the allocation and use of resources. There has been inadequate awareness that better use of timely, accurate, and relevant information can have significant ramifications for advancing health [23].

Whereas there is evidence that tasks shifted to community level workers can be undertaken effectively [15,18,11], perspectives on tasks selection, and CHW motivation among various stakeholders in the Kenyan context have not been described. Franco et al [14] suggests that non-financial incentives are successful in motivating workers, yet these strategies have not been described in different Kenyan contexts. This paper seeks to describe the perspectives of various stakeholders on task shifting to CHWs as well as sustainable motivational strategies for them. The paper demonstrates motivational strategies that were perceived to be appropriate to retain CHWs in different socio-demographic contexts in Kenya, in the context of task shifting, including data collection, analysis, and use in decisions and actions for health improvement in the different contexts.

## Methods

The study was analytical and comparative in design using qualitative methods of data collection. The study compared task shifting and motivational strategies in three different socio-demographic sites: a high density peri-urban informal settlement (slum), a sparsely populated nomadic site, and a rural agrarian site.

### The setting

The study was undertaken in four purposively selected health units in rural agrarian, nine in peri-urban and two in nomadic sites. The four rural agrarian Community Health Units included in the study were situated in Butere District, Western Province, while peri-urban and nomadic sites were situated in Kisumu town Nyanza Province and Garissa, North Eastern Province, respectively. All selected Community Health Units were part of the community health strategy scale-up program, in partnership with the Ministry of Public Health and Sanitation.

### Sampling

In the Kenyan community health strategy local populations of 5000 were served by 30 CHWs each taking care of 20 to 50 households. However, CHWs in the peri-urban areas served up to 100 households due to high density of households. Four health facilities were selected in Butere, and two in both Nyalenda and Garissa. All community health units served by these health facilities were included in the study. The idea of qualitative research is to purposefully select informants that will best answer the research question [24]. The sample size was not considered an important factor in this study, since according to qualitative researchers; the intent is to continue data collection until data saturation, a point at which no new information is being generated, as described by Denzin & Lincoln [25].

Study sites were purposively selected to represent contrasting areas of Kenya: nomadic, agrarian rural and peri-urban. Background information is summarized in Table 1.

**Table 1:** Background characteristics of study sites

Sites	Nyalenda	Butere	Garissa
**Context**	Peri-urban slum	Rural agrarian	Rural nomadic

**Location**	Kisumu county, Nyanza Province, Western Kenya	Kakamega county, Western Province, Western Kenya	Garissa county, North Eastern Province

**Population density**	663.9 people per km^2^	154 people per km^2^	18 people per km^2^

**Mean distance to health facility**	<2km	2-5km	>50km

### Data collection

Data collection involved focus group discussions and key informant interviews. Ten key informant interviews were undertaken at the national level, four in each of the three study districts, eight with service providers in the rural site, and two in the nomadic and urban sites. Focus group discussions comprised two youth, two female health consumers, two male health consumers, and two community health workers for each of the eight health facilities included in the study from the three different study contexts. Purposive sampling was used to identify the knowledge rich individuals, usually heads of various structures in the health sector, from national to local levels.

A common set of guiding questions was used at all sites^i^.

### Data analysis

The qualitative data from the focus group discussions and key informant interviews were transcribed, anonymized, and coded using content analysis framework as described by Miles, M. B. and Huberman A. M. [26]. From the themes and sub themes, common emerging issues and concerns were identified and narratives were constructed. Transcripts were read and reread to identify emerging issues and themes by site-specific teams first. Thereafter cross-site comparisons were made. Responses were compared across type of respondents (consumers, CHWs, health providers, and managers), and study sites. Narratives were constructed on views of various stake holders on what tasks can be shifted to community health workers and what strategies are appropriate for motivating them in their work.

Ethical approval for this study was granted by the Moi University Ethical Review Board.

## Results

### Description of task shifting

Policy makers and partners described task shifting as “the process in which certain services that ought to have been offered by the superior persons are delegated to less qualified persons,” as stated by a policy maker at the national level. Managers viewed it as tasks that can be performed by lower cadres such as CHWs. Community health workers described it as “tasks performed by nurses lowered to be performed by CHWs,” stated by a CHW focus group discussant. While consumers understood it to mean “shifting activities from the health facilities to the community” to be carried out by CHWs, according to a female consumer focus group discussant.

### Tasks proposed to be shifted to community health workers

There was agreement at all levels and sites that all promotive and preventive services were appropriately shifted to CHWs, according to the Kenyan policy guidelines, since they were close to households and were best placed to communicate with them within the context in which they lived their lives. Concerning curative services, there were differences in perspectives by categories and levels of respondents, and by study site. Policy makers emphasized that shifted tasks should include home based care for the chronically ill; managers at the district level did not support going beyond preventive and promotive care in task shifting; CHWs were interested in taking on a range of curative services. This was particularly strong in peri-urban and nomadic contexts. The rural CHWs mentioned only promotive and preventive services, for which they are currently trained. Consumers’ views supported their CHWs, emphasizing addition of curative tasks in peri-urban and nomadic sites. In addition, CHWs and consumers from all sites mentioned data collection, see table 2.

**Table 2:** Proposed curative and data tasks that can be shifted to CHWs

Context	Tasks that can be shifted
**Nomadic**	Home care for malaria and pneumonia Acute respiratory infection (ARI) Epidemic recognition and reporting Home based care for HIV/AIDS (nursing care, feeding, psychosocial support, treatment of infections) Hospice services for cancer and stroke patients Community-based rehabilitation for chronically impaired (physical and mental) Arthritic care Heart conditions Community-based nutrition Direct observation treatment for TB/AIDS Household data collection

**Peri-urban**	Home care for malaria Acute respiratory infection Epidemic recognition and reporting Home based care for HIV/AIDS (nursing care, feeding, psychosocial support, treatment of infections) Hospice services for cancer and stroke patients Community-based rehabilitation for chronically impaired (physical and mental) Arthritic care Community-based nutrition Direct observation treatment for TB/AIDS Injections Household data collection

**Rural**	Home care for malaria and pneumonia Acute respiratory infection Epidemic recognition and reporting Home based care for HIV/AIDS (nursing care, feeding, psychosocial support, treatment of infections) Community-based rehabilitation for chronically impaired (physical and mental) Community-based nutrition Direct observation treatment for TB/AIDS

**Research tasks shifted**	Data collection Data cleaning Data analysis Data interpretation Data dissemination Conducting dialogue sessions

It is noteworthy that when respondents were asked to propose tasks to be shifted, all CHWs and consumers, but not policy makers or managers, in all three sites included data collection, analysis, and use of data for dialogue. “The analysis of data, display on the chalkboard and use for dialogue has enhanced ownership of data by the community,” said a community health extension worker. CHWs, Community Health Committees (CHC) and managers cited the importance of involvement in community-based data collection for health education, dialogue, and decision making, at all levels of the health system, starting with the household.

The majority of respondents wanted CHWs to be responsible for dialogue at the household level, but also recommended that care givers be trained on data use. This implies that CHWs should manage regular household data collection and spear head evidence-based dialogue sessions at the community level, using the data. This should be backed by a records and referral system. Asked about sources of information for dialogue, the community-based information system was the most frequently mentioned. Respondents also cited the use of information in other tasks such as health education on family planning, reproductive health, maternal health, child health, immunization, malaria and fever, and use of insecticide treated nets, worms, water and sanitation, general and personal hygiene, HIV/AIDS care, food and nutrition, and many other elements of the essential package of health care. Most used data monthly for this purpose. The indicators were selected by the community and approved by the ministry of health.

CHC recommended refresher courses for CHWs on valid and reliable data, so that the data collected reflects the true situation at the population level. They also felt that they should be trained on data analysis and be given equipment to process data as this would improve the frequency and triangulation of data from different sources. A Community Health Committee member expresses it thus: “increase the number of CHWs to complete the coverage, train us on data analysis, provide equipment like photocopier, laptop, desktop computers to support data collection and processing to improve on the frequency of data collection, monitoring and evaluation. Triangulation of data from different sources, would improve quality of decisions based on data.” They wanted the CHC to take greater responsibility for data, to share it with policy makers at the local level to influence decision making like improving on security, building more toilets, and bringing more piped water to the community.

### Tasks taken up by CHWs beyond current policy guidelines

The study found that there were a number of tasks that were being performed by community health workers which were beyond the range provided for in the current policy guidelines. There were similarities and differences in the types of such tasks and the reasons the community health workers were performing them in the three sites.

In the nomadic site, it was evident that CHWs were undertaking some curative tasks such as treatment of malaria and pneumonia, and dispensing of medicines. This was in response to demand. In this context, health service accessibility was limited by both geography and civil unrest, for large segments of the population. Community health workers described the necessity of modifying their care package for hard-to-reach populations; a consumer in Garissa had this to say: “at times we try to access the [government health] services but the resources are not there and so we have to opt for other health care alternatives rather than die.”

Similarly, in peri-urban sites, the community health workers had expanded their care package. The reasons for doing so were different from the nomadic site. Although the respondents felt that they were responding to the demand from the community, they were more strongly driven by desire for income, and people were more willing to pay them for curative rather than preventive services. They argued that their levels of education were adequate for them to be trained for such curative skills that they could sell to the community. A peri-urban CHW said “life is too expensive and working for free is not working for us, we need to compliment the free services with the skilled curative services for a certain fee.”

**Table 3:** Tasks mentioned by different stakeholders as informally taken up by CHWs

Stakeholders	Curative Tasks taken up informally by CHWs
Policy Makers	Home based care for the chronically ill

Donors	Dispensing of drugs (ARVs), anti-malarials, dressing of wounds

Managers	None

Nomadic CHWs	Treating diseases, deliveries, injections

Peri-urban CHWs	Circumcision, delivery, injection, immunization, blood tests and treating diseases.

Rural CHWs	None

Nomadic Consumers	Injection, deliveries, drug dispensing, treating diseases

Peri-urban Consumers	Injection, family planning, circumcision, deliveries, drug dispensing, dressing of wounds

Rural Consumers	None

### Motivational strategies in ensuring quality

A number of motivational strategies were mentioned by different categories of respondents.

#### Training and career path progression

Respondents expressed the need for in-depth training on selected curative services, some of which they were already undertaking but for which they had not been trained. The CHWs emphasized the need for formal training as part of motivation to ensure their retention: “new cadre to be created CHWs should be trained and should have their own cadre,” “Supervision should be reinforced by the person who is the in charge of the facility, meeting CHWs and discussing the topics they have.” The consumers agreed with the need for frequent in-service trainings. Policy makers supported the idea of a career path that requires that CHWs are admitted for further training based on guidelines of professions of interest, criteria for recommendation to progress, and performance, which is measured by the data they collect.

Managers supported the idea of career path. A district health management team member said: “There should be a chance for them to climb up. They have desire to grow up so they should be allowed. A scheme of service that provides for career progression for CHWs. This can be done through looking at their work, those people who are working hard should be given that priority so that may be motivated. CHWs have expressed a strong desire to grow so they should be given an opportunity.” They further suggested that the government should provide a scheme of service that provides for career progression for CHWs. This can be done through looking at their work, improvement in health indicators that they have brought about, based on regular household data collected, to give priority to the champions. They felt that financing of the community health strategy at the county level is crucial for the success of the program. Another remarked: “Financing of the community strategy at the municipality is key.”

Respondents found training as key: “you know in the world of today you just need to know your job well if you don’t you are ridiculed in the society, that means you lose the respect and you lose market, it is good if we are trained then people respect our jobs, they know we are experts in it, it feels good when you are known to do your job well,” stated a rural respondent. It emerged that peri-urban CHWs wished to acquire skills to sell, because of hard economic times in the country. The respondents in both the peri-urban and the nomadic sites said it was important to be trained frequently and given refresher courses, given that new trends and disease patterns emerge frequently.

The respondents insisted that training was the backbone of the task shifting concept. A respondent from Garissa (nomadic) said “because of the critical work we are forced to do then it is mandatory that we are offered training as new things emerge especially in our area where access to health services is very hard, when we are trained we are able to save lives especially of mothers and small children, without training we offer services of lower quality that endangers people’s lives, so it is important when we trained frequently.”

The respondents from the rural context stressed that because of the low literacy levels, health workers at the community level needed frequent training. The community health extension workers strongly recommended frequent refresher courses for CHWs to reinforce their confidence in carrying out their duties, hence earning respect from the community. One consumer said “when I know that the community health workers are trained frequently on their job, then appreciating their advice on health issue becomes a little easier, if they are not trained and they start giving health education and some of them have not gone to school, I really doubt the expertise, so it is good if they are trained then we as a community we are able to believe them.”

#### Accreditation, Regulation and Licensing

Policy makers recommended devolution of responsibility for CHWs to the county and ward, for them to hire their own CHWs, motivate and supervise them. Supported by a functional referral system. They stressed the importance of supervision and regulation of their work by establishing a Community Health Strategy District Management Structure, with a focal person whose sole job is to coordinate their activities. “They should make sure they monitor and evaluate the community interventions in their district,” said a policy maker. They expressed the need for a context specific regulatory mechanism. “This is very critical and must be devolved to county and ward levels to enable the necessary context specific reward.” Further, policy makers recommended an accreditation framework: developing a consensus on context specific tasks, required competencies, courses content, approved by recognized academic institutions as well as guidelines on pre-service and in-service components. This would permit certification, regular review, and a licensing mechanism. Community level support mechanisms would include community health extension workers, who is a trained health professionals deployed at the community level, reporting to the health facility in charge, to supervise the CHWs. The CHWs supported the need for formal training, regulation, registration and annual licensing, supervision, and linkages to the health facility to ensure their retention. They also stressed the importance of good relations with health facility staff and mutual respect.

The respondents from the nomadic area were of the view that if the community health strategy support structures were adequately supported, they would provide an enabling environment to carry out their duties diligently “when this structures are not functioning you are not sure sometimes whom to report to depending on the weight of the matter.” explained a community health worker. The structures would then provide a proper regulatory mechanism for CHWs in the process of carrying out sensitive work, especially in the nomadic site where circumstances have forced them to offer curative services.

In the peri-urban setting, they strongly supported the need for regulatory structures to guide task shifting, particularly curative and data collection activities. The respondents reported that this would make them feel confident and have a sense of belonging because they would have a back up. The health facility committee felt that the structures and regulatory mechanisms would prevent unauthorized individuals from misusing their names. They felt that “this would save lives,” according to a consumer.

The rural context respondents concurred, stating that regulatory structures would make the community gain confidence in their work, thus strengthening their relationship with the community. A respondent said: “at least we will have a place where we can report issues of concern, but if they are not there we do wrong things sometimes because we know we are not accountable to any one, and by this the community loses trust in our job.” This came up strongly in the shifting of data management tasks, especially during dissemination. The community felt that data collected and disseminated needed to be regulated by the governing authority because they were supposed to communicate factual information about the health status of a given community. Community health workers felt that recognition by both the health facility and the community was very important.

The issue of licensing emerged from CHWs, community health extension workers and community health committees especially in the nomadic and peri-urban areas. They felt it was necessary to reduce unauthorized individuals masquerading as CHWs. There are people who are opportunists and pose as health workers yet they are not trained: “it feels so bad when one poses like a community health worker yet they are not and in Garissa sometimes they give wrong medication so that they get money, it is very bad, lives can be lost the Government need to regulate this by giving licence for those who have proper skill, this happens because we are not able to access health services and sometimes you don’t find medicine so they go for those who they think can do it and sometimes they go to wrong people! It’s very bad,” said a CHW in Garissa.

In the peri-urban area, the respondents said it is good if an in depth training is offered and given the licences to operate: “we know how it works in the slums and we know what can be appreciate so we need to be trained and given licensed to give services, some people shy away to go to hospital because they don’t have enough money , not to mention transport, for us we can offer at the door step, this we can help a dying child or a sick woman, when we are licensed we feel we are recognised and appreciated that we can exercise the skill that we have learnt.”

The rural respondents expressed the need to be trained first before anyone is licensed to do any tasks that have not been delegated to the community. One community respondent said “as consumers we rely on their services so if they are to maybe allowed to treat disease and we are not sure if they were trained and certified then it can be a disaster if they do the job in wrong manner.” Licensing was said to be a good idea but only if the work was beyond the preventive and promotive tasks that have already been shifted to community, such as home based care and treatment of malaria.

#### Supervision

Service providers mentioned that close supportive supervision by health professionals and the community health committees was a critical component in their hardship context. Support supervision and linkage emerged strongly and they reported that they are proud when they are linked to a health facility: “we are able to learn a lot and people respect that our work is credible, but if you are not supported by anyone or linked to any recognised body, people, especially our clients, don’t recommend your work to the community, so it is important to be supervised, supported and linked to a recognised body in the process of service delivery to the community.”

The rural respondents noted the need for close supervision: “most of us are primary drop outs so these things require a lot of close supervision and support in order to offer quality service especially in research work, it needs a lot of reasoning.” They suggested that the Ministry of Health should improve infrastructure up to the dispensary to strengthen the community health units.

#### Resource allocation

Resource allocation by the Ministry of Health to community level care emerged as a strong factor in all three contexts. In the nomadic setting, the respondents said that the main driving factor was the availability, accessibility, and equity in allocation of the resources, including data collection tools needed to do their job. The respondents said that it was the driving force for service provision, “We don’t mind even if we are not paid, what is more important than anything else is when we have the equipment to work,” CHW from the nomadic site.

The peri-urban respondents expressed the need to be allocated resources for effective provision of services, as one said “When there is equitable resource allocation, we enjoy our work, but if the resources are not allocated we feel very frustrated even the community look down on us, it’s very frustrating and shameful, we lose confidence” and a second respondent said “When we have the necessary equipments it motivates us and we take pride in our work.” This view was strongly supported by the community health extension workers: “We supervise work at community level it becomes very frustrating when volunteers at community level are not able to do their work.”

In the rural setting, the health facility committee and the community health committees strongly recommended allocation of resources for the community health workers. The reasoning was that preventive measures were more effective in stabilizing the health status of the community. They felt it was a cost effective way of preventing and controlling communicable diseases. CHWs explained further that resource allocation was critical to efficient service delivery, especially in health education data management tasks.

#### Recognition and identification

Respondents expressed that badges, uniform, card, and certificates, depending on the skill acquired, was a crucial motivation component in task shifting of health services. In addition, educating the community on the benefits of CHWs’ work would help.

In the nomadic site, the respondents said that when “we have the badge that has been certified by the Ministry of Health then it becomes easier to offer curative services in particular.” Having a badge would regulate the service provision of the CHWs at the community level, this would ensure that the work is done only by those who have been trained and have qualified to do the job, by making them recognizable by the households and authorities. This was strongly supported by the community health extension workers, the health facility committees, and the community health committees. They viewed it as an effective regulatory mechanism in ensuring quality.

The peri-urban CHWs expressed the need to regulate the market by awarding certificates depending on the level of training, not just a general one. They expressed the need for the badge especially when selling their skills to the market. Their emphasis was on the curative services. On data management tasks, they said that they would be more motivated if they received recognition that they had completed the training in data collection, analysis, interpretation, and dissemination of findings. They said the community normally wants to verify if indeed they are collecting data that will be of any use to the community.

The rural CHWs’ views were not much different from the other contexts. They felt that having badges and certificates was a strong motivator. They said that it would boost their self esteem and morale for work. The health facility committee supported the view by saying it would control the attrition rates. They explained that certificates should only be given to those workers patient enough to complete the course, and regularly involved in household registration and dialogue, that deserve recognition. Community health extension workers recommended the badges, especially when CHWs are referring patients to the health facility or when they are tracking immunization, TB, and ARV defaulters. “When they go out there to do such kind of work, it boosts their confidence and they do with authority because they have been certified by recognized authorities” said a respondent. They added that the certificates and the badges should be awarded strictly by the Government of Kenya through the Ministry of Health.

They mentioned the importance of support, especially from the Government side “You know when you’re supported by the Government that is security on its own, you earn respect for whatever kind of job you do, but if it is not there like the way sometimes we experience it, there is no much respect from the community sometimes you are even harassed by the community itself.”

**Table 4:** Comparison of different motivation strategies by different respondents.

POLICY MAKERS	MANAGERS	CHWs	CONSUMERS
Devolution to County Supervision Compensation Linkages Regulation Accreditation and regulation Supportive mechanisms Referrals	Training Supervision Linkages to the health facility	Training Supervision Regulation Linkages to the health facility Registration and annual licensing Good relations with facility staff and respect Introduction of a new recognized cadre of trained CHWs Referrals Frequent dialogue sessions with health stakeholders	In service training Transport facilities Linkage to health facility Provision of equipment Constant health education

Well defined career path for the CHWs: Sustainability of task shifting Composition of CHWs: Tangible financial reward.	Career progression Financing	Career progression Financing Motivation: Lunch transport Cover direct costs, Providing bags, Provide protective equipment, Rewards, Appreciation.	Frequent in service training, Transport facilities, Provision of equipment and supplies Recognition Identification (badge, uniform, card) Educating community

#### Incentives

The policy makers recommended some form of context-specific compensation to retain CHWs. This could be in the form of regular pay, output-based reward, or through community-based organizations. Additionally, households could contribute regularly to the support fund. The view of CHWs was that they would be motivated by such things as lunch, transport, covering direct costs, providing bags, bicycles, protective equipment for community-based care, recognition of output through in kind rewards, and tokens of appreciation, based on data. Consumers concurred, emphasizing the need for transport to facilities, provision of equipment and supplies.

In all three sites (nomadic, peri-urban, and rural), respondents supported a range of support items needed in the process of task shifting, especially in the curative and data management tasks.

The peri-urban respondents reported that the hard economic realities in their context affect the quality of their work. The respondents stressed that services that were curative, but approved by authorities, needed some incentives or compensation. One said “life is expensive you can’t volunteer to treat someone using your own money and the client gives nothing in return you can’t sustain the work, something has to be paid in return to sustain the job!” They also felt that data collection tasks that had been shifted to the community level needed some monetary compensation. They reported spending a lot of time in training, data collection, and the final analysis, not to mention dissemination during the dialogue process. From the discussion, it emerged that data collection tasks should be compensated in order to maintain high quality of data.

In the nomadic area, the health workers cited the need for transport and said that in order to cover their clients, at times they are forced to use their money. They were of the view that some transport allowances should be allocated to them. They stressed that compensation was a driving force in the health service delivery, one respondent said: “When am to treat minor ailments like fever or malaria, I need the kit that is fully equipped and the only way to afford that is I sell my services in order to make it running if I don’t do that I will lack necessary resources to work with.”

The rural community health workers expressed the view that compensation was a strong motivating factor, especially on data collection tasks. One of the respondent said “during trainings it’s as if you have gone back to class, it drains you mentally, you think very hard to understand this things so a little token is not bad so as to motivate us it keeps us going, the work for sure is heavy.” And another said “Best way of retaining is to pay them as regular workers but pay them from the community fund. In own set ups transform Community Health Units into Community Based Organizations and save funds from which they can borrow.”

#### Management of community-based health information systems

It emerged that motivation can help improve data quality: “to improve data quality reduce the number of households to CHWs, tools should come in time. Before data is collected awareness should be done at community meetings for the community to give the correct information. Give drugs kit to increase the credibility of the CHW.” Motivation could increase, if CHWs were more listened to, and better involved in decision making. “We should carry drugs, painkillers, and lotions to motivate household members.”

Respondents further expressed the fact that motivation comes from improvement resulting from evidence-based dialogue held with their households and communities regularly, such as immunisation status, latrine coverage, ANC attendance, family planning, skilled delivery, nutrition status, use of treated water, use of insecticide treated nets, and effective monitoring and evaluation of their own progress. Asked about lessons learnt in community-based information management, they mentioned increased community interest in evidence; having health talks based on the real situation. “It makes people make the right decision when planning for the community, people air their views on problems at the right time, information shows what communities need, it improves effective communication of health messages.”

They felt that access to important information had been made easier; making people change attitudes and practice. “You can reach all the people in the community with critical information such as about outbreaks monitoring; it brings stakeholders together to dialogue, data gives direction on intervention to tackle health problems.” Household information displayed by the community health workers supported by community health extension workers provided immediate feedback to the community leading to relevant health education. It tracked actual conditions on the ground during dialogue day, involving other partners to share their data and promote healthy competition towards health improvement. “It is a good thing to use data for dialogue, data reflects the health status of the community, clients can be referred and action taken. Community members come together to agree on objectives that are then tackled towards health, improvement of health. It gives the real picture of the Community Health Unit.” Most participants agree with the decisions made by the group. “People motivate each other and work together. All participants come together and contribute,” explained a community health extension worker.

## Discussion

Our findings suggest that all categories of respondents agreed promotive, preventive, and simple curative tasks can be shifted to CHWs, as provided for in Kenyan policy guidelines [27]. The difference is in the range of curative tasks. The policy makers see the need to expand the role of CHWs to include some curative tasks, especially home-based care for the chronically ill, as well as community-based data management. Community health workers and consumers from peri-urban and nomadic settings suggested the inclusion of curative care. This view is supported by research and experience from other countries in Africa and beyond [28]. The Health Systems Report [26] further indicated that evidence on CHWs from The Gambia, South Africa, Tanzania, Zambia, Madagascar, and Ghana were not only cost-effective, but also enhanced the performance of community level health programmes.

The expansion of the service package provided by CHWs in the hard-to-reach nomadic households, to include health promotion, disease prevention, basic curative care and referrals, monitoring of health indicators, and creating vital linkages between community and formal health systems, where the human resource crisis is at its worst, has been supported by many researchers[1,3,5,9,11,12]. Thus, their role emphasizes their technical and community management function [3]. Therefore, CHWs can play a crucial role in broadening access to and coverage of health services in remote areas.

An evaluation conducted by Pakistan’s Ministry of Health enumerated the successes of the Lady Health Workers in line with health improvement [4]. Hence renewal of interest prompted by the AIDS epidemic and the failure of the formal health system to provide adequate care for people with chronic illnesses [29].

In this study, district health managers were unconvinced about the need to expand the CHWs’ tasks to curative care. Their view was consistent with the views of rural CHWs and consumers. They were content with the policy as is: mainly preventive and promotive tasks, but with treatment of minor ailments, and first aid, for which they need to be well trained, supported, and supervised. Yet there is lack of equity and efficiency in the provision of health services that would have been able to prevent deaths caused by preventable diseases [30]. There is evidence that CHWs can play an important role in helping to achieve the Millennium Development Goals for health, particularly for child survival and treatment of TB and HIV/AIDS if allowed to take on some curative tasks [1,11,15,18], supported by appropriate regulatory mechanisms.

In this study, nomadic CHWs and consumers viewed the expansion of tasks to include curative aspects, beyond what is provided for in the current policy guidelines, as inevitable, given the great unmet demand, and grossly inadequate access to essential care. In their view, expansion is a must, not a choice, as supported by other workers [10,1]. The emphasis on inclusion of curative care among CHWs’ tasks in nomadic areas is supported by the fact that this area lags behind in improved geographic access to health care services. An analysis of health care coverage in Kenya by Noor AM *et al.*[31], demonstrated that over 80% of the population in nomadic areas live beyond the five kilometre distance from a health facility, required by the Ministry of Health. A systematic review by Chopra and his co-workers concur with the findings of this study supporting the role that lay health workers may play in extending essential health services to ‘hard-to-reach’ groups and areas; and in substituting for health professionals for a range of curative tasks [32].

In the peri-urban site, the community health workers tended to have higher levels of education than the other two sites. They tended to be more interested in career development, hence their desire to develop into professional health workers with competencies and services to sell to their consumers, given the harsh economic realities in urban slums.

Policy makers in this study stressed the importance of supervision and regulation of CHWs’ work by establishing regulatory, technical, and logistical support mechanisms at county levels. Other workers have concluded that in order to carry out their tasks successfully, CHWs need regular training, supervision, and reliable logistical support [33]. Non-financial incentives and human resource management tools have been shown to enhance health worker retention in Africa [34]. Further, policy makers recommended an accreditation framework, based on specific tasks, required competencies backed by certification, and licensing mechanisms. Important constraints included inadequate supervision; insecure funding for incentives, equipment, and drugs; failure to integrate CHW initiatives within the formal health system; poor planning; and opposition from health professionals [35].

The recommendation by policy makers to devolve the responsibility for CHWs to the county level provided for in the new Kenyan constitution has been supported by other workers [36]. The nomadic area respondents were of the view that if these structures were adequately supported, they would facilitate the work of CHWs in their work, especially in the nomadic site where circumstances have forced them to offer curative services. If the structures are put in place and operational, they would provide proper regulatory mechanisms for CHWs. In the peri-urban setting, they strongly supported the need for regulatory structures to guide the process of task shifting services, particularly curative and data activities. Community health workers felt that recognition by both the health facility and the community was very important, to prevent misuse of their name by other non-trained individuals.

These findings strongly suggest an urgent need for context specific regulatory mechanisms to be put in place, including accreditation of training, registration, and licensing. Unmet needs, and a huge demand for health care services, attracts suppliers such as CHWs, some of whom could begin functioning as unlicensed health care providers. Data collection, analysis, and reporting offers a mechanism of regular contact with the service system as well as a framework for regulation, hence the need for urgent policy review. Other studies have demonstrated that increased demand either due to increased population, distance, or prevalence of disease, tends to lead to task-shifting, formally or informally. In Uganda, task shifting in HIV/AIDS has been widespread, with the involvement of CHWs in the care and support of patients. This has been a success, particularly where tasks are shifted from the health facility to patients’ relatives and community workers [37].

Peri-urban CHWs had strong feelings that they live in an economically precarious context, since inhabitants have moved away from subsistence economy in the villages and must earn their livelihoods in the urban cash economy. In their context, volunteering is not a viable option. Given the population density, they realize there is demand for curative services that they can respond to. They see their role in data collection as a means to an end. It gives them the information they need to identify clients and to gain access to households.

Informal task shifting has been described in other settings. Swaziland, for example, does not have a national policy on task shifting, yet task shifting occurs informally throughout the country. Communities have reported positive results, including increased uptake of prevention of mother-to-child transmission services, increased immunization coverage, and a reduction in the number of defaulters on treatment for tuberculosis [38].

Studies have shown that continuous training is a powerful motivating factor, as mentioned by CHWs and consumers in this study, and described by Kaseje and Kaseje [7]. They found that in a rural setting, volunteers thrive when they are regularly given ongoing training, life-long, in addition to supportive supervision, as well as being given the tools required for their work.

## Conclusions

From these findings, task-shifting and motivational strategies need to be context specific, hence the need to devolve the responsibility and resources to local government levels.

The training of the community health workers should address the required context specific competencies, but should be appropriately accredited, certificated, and recognized. Inclusion of curative tasks for CHWs, particularly in nomadic contexts, is inevitable but requires accreditation of their training and regulation of their tasks.

A regulatory mechanism is urgently required, that should be adjusted to specific contexts, linked to local governing and management structures, while providing opportunity for career progression.

## List of abbreviations

ANC: Antenatal clinic; ARI: Acute respiratory infection; ARV: Anti-retroviral; CHC: Community health committees; CHW: Community health workers; DFATD: Foreign Affairs, Trade and Development Canada; GHRI: Global Health Research Initiative; IDRC: International Development Research Centre; TB: Tuberculosis; WHO: World Health Organization

## Competing interests

The authors declare no competing interests.

## Authors’ contributions

BMO supervised data collection, spearheaded data analysis, and synthesized the contributions of the other authors into the first draft of the manuscript. She further engaged actively in the revision of the manuscript in response to critiques from internal peer reviewers. EA participated in data analysis and drafted the section concerning the nomadic context. NE developed analysis framework, participated in data analysis, and carried out technical critique of the manuscript. DM and LM analyzed the data from rural and peri-urban sites, respectively, and drafted the respective components of the manuscript. DCOK designed the study, management the research process, supervised all aspects of the study and the team members, participated in the analysis of data, synthesized the contributions from other authors into the manuscript, and took the lead in editing the manuscript based on internal peer reviewers’ comments. All authors read and approved the final manuscript.

## Endnotes

^i^ The main guiding questions included: Describe any policies on Task Shifting in Kenya that you are aware of. To what extent is task shifting being implemented? In delivery of healthcare what tasks have been or can be shifted to community level? What are your views on the current practice of task shifting to community level workers? Describe ways in which task shifting can best be implemented in the provision of health services at the community level. In your view, how can task shifting be guided and regulated? How is task shifting influencing health service delivery? How can Quality be assured in task shifting? What is the referral system linkage for the community-based workers? How are the workers trained? What are your views on how task shifting can be sustained at the community level? What is the career path for the Community Health Workers? What are your views on compensation of community workers? What do you think can be done to retain the community workers?

## Declarations

The publication costs associated with this article are funded by Foreign Affairs, Trade and Development Canada and the International Development Research Centre through the Global Health Research Initiative.

This article has been published as part of *BMC Health Services Research* Volume 14 Supplement 1, 2014: Uptake and impact of research for evidence-based practice: lessons from the Africa Health Systems Initiative's research component. The full contents of the supplement are available online at http://www.biomedcentral.com/bmchealthservres/supplements/14/S1
